# Miniproteins may have a big impact: new therapeutics for autoimmune diseases and beyond

**DOI:** 10.1038/s41392-024-02010-z

**Published:** 2024-10-28

**Authors:** Nariaki Asada, Christian F. Krebs, Ulf Panzer

**Affiliations:** 1 III. Department of Medicine, University Medical Center Hamburg-Eppendorf, Hamburg, Germany; 2 Hamburg Center for Translational Immunology, University Medical Center Hamburg-Eppendorf, Hamburg, Germany; 3 Hamburg Center for Kidney Health (HCKH), University Medical Center Hamburg-Eppendorf, Hamburg, Germany

**Keywords:** Adaptive immunity, Translational immunology

In a landmark study recently published in *Cell*, Berger et al. demonstrated that computationally designed miniproteins could serve as novel, orally available treatment strategies by specifically targeting cytokine signaling pathways through the potent inhibition of ligand-receptor interactions in the body.^1^ This breakthrough study unveils the potential for miniprotein-based therapies to revolutionize molecular treatment strategies for autoimmune diseases and cancers.

A better understanding of the molecular mechanisms underlying autoimmunity and cancers has paved the way for the development of new therapeutic agents, including mAbs and small molecule inhibitors, which specifically target critical molecular pathways. These targeted treatment options are essential because treatment with broad, non-specific agents could be avoided, thereby reducing undesirable adverse effects, including systemic immune suppression. For instance, mAbs targeting cytokines such as TNF-α, IL-17, or IL-23 have significantly improved the management of autoimmune diseases, including rheumatoid arthritis, psoriasis, and inflammatory bowel disease (IBD).^2^ Small molecule inhibitors have also shown efficacy in many conditions. Oral Janus kinase (JAK) inhibitors, for example, have been approved for inflammatory diseases, including IBD. Additionally, oral antagonists targeting IL-17 and IL-23R are currently in clinical trials, potentially offering more options for future treatment.

However, despite their efficacy, these targeted treatment options present significant challenges. mAbs require high production costs and the need for parenteral (injection-based) administration, making them less convenient and accessible for patients. Moreover, some patients develop anti-drug antibodies, causing a reduced response to treatment. Small molecule inhibitors are less expensive and less likely to induce the development of anti-drug antibodies. However, their lower specificity can lead to off-target effects, and severe side effects are reported. Thus, there is an urgent need for innovative approaches that efficiently translate immune profiling discoveries into safe and more accessible treatment options.

To address these issues, Berger et al. implemented a workflow to computationally design, produce, and validate miniproteins that efficiently bind to cytokines or cytokine receptors. Their manuscript describes different miniproteins targeting IL-23R and IL-17A, which are well-established molecular targets in autoinflammatory diseases, including psoriasis and IBD.^2,3^ Miniproteins have several advantages over monoclonal antibodies. First, miniproteins exhibit extremely high affinity in the low picomolar range. Second, they demonstrate remarkable stability and resistance to heat, acid, and proteolysis, making them resilient to degradation in the gastrointestinal (GI) tract. Third, due to their small size and stability, they can be administered orally and absorbed through the gut, ultimately reaching the systemic circulation. The half-life of the miniproteins was shown to be relatively short. Given their small size and stability, this could be attributed to excretion *via* the kidneys and/or liver. Despite this short half-life, miniproteins are expected to retain sufficient efficacy due to their extremely high affinity and slow dissociation rate, which enables sustained target saturation. Their oral bioavailability represents a paradigm shift in therapeutic strategies because it could significantly improve accessibility to treatment and patient’s quality of life. Moreover, higher specificity and fewer side effects are expected for miniproteins compared to small molecule inhibitors, providing safer treatment options in the future.

The authors validated the effect of the designed miniproteins in in vitro studies, highlighting that miniproteins can efficiently block IL-23 and IL-17 signaling in human cells. Moreover, they tested the miniprotein targeting IL-23R in a preclinical model of IBD using humanized mice, where its once-daily oral administration resulted in significant improvement in clinical scores. The efficacy was comparable to that of a clinical mAb (guselkumab), demonstrating that miniproteins could offer a more convenient and potentially cost-effective alternative to mAbs, with the added benefit of oral administration. This finding is particularly noteworthy because it suggests that miniproteins could enhance patient compliance and accessibility, reducing healthcare costs. In addition, the designed miniproteins might be less immunogenic. Because of the stability at low pH, resistance to proteases, and high solubility, miniproteins are less likely to be taken up, digested, and presented by antigen-presenting cells, which would reduce the risk of anti-drug antibody production in patients.

De novo design of miniproteins using computational methods has increasingly attracted attention. Miniproteins, theoretically, can be designed against any proteins with known three-dimensional structures. The computational design of protein-protein interactions has a long history. Still, only recent advances, such as protein docking-based methods (also reported by David Baker’s group in *Nature* in 2022), have made designing miniproteins against any protein structures possible.^4^ Deep learning-assisted methods enhanced the binder design and yielded nanomolar-affinity or better binders directly from computational pipelines. The authors also began the design process with the structure of human IL-23R in complex with IL-23p19. The miniproteins were optimized through yeast display libraries and deep mutational scanning techniques. These design and optimization processes allowed the researchers to fine-tune the miniproteins for maximum efficacy and stability.

**Fig. 1:**
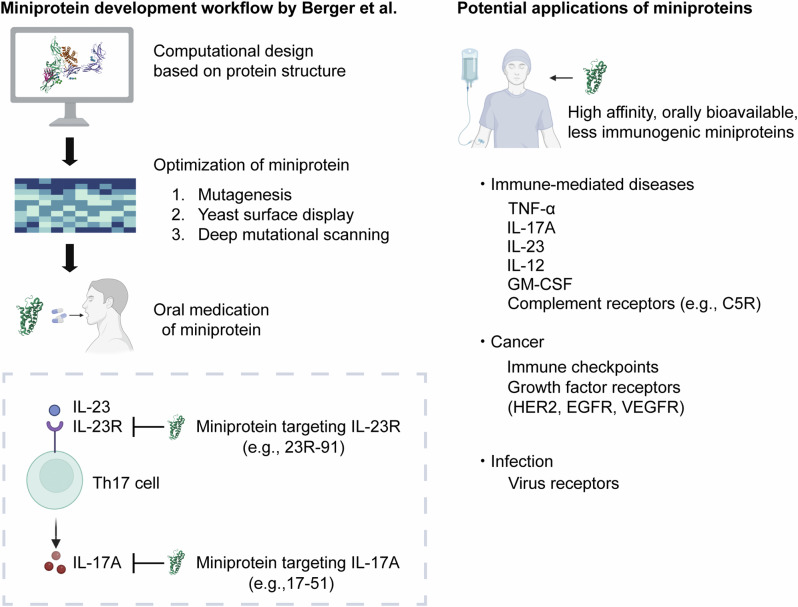
Miniprotein design and potential applications. Miniproteins are designed based on protein structure information and optimized to enhance their affinity and stability. The optimized miniproteins can exhibit high affinity for their target proteins, be orally available, and show reduced immunogenicity. Miniproteins can be developed to target various ligand-receptor combinations, which play key roles in immune-mediated diseases, cancer, and other conditions

The significance of this research extends beyond the treatment of IBDs. Another study from the same research group reported that miniproteins can target innate cytokine signaling by blocking IL-1R1 or IL-6R, potentially ameliorating cytokine-mediated tissue damage.^5^ Moreover, recent advancements in sequencing technologies, such as single-cell RNA sequencing (scRNA-seq) and spatial transcriptomics, have made immune profiling across various diseases possible, unraveling the critical roles of different cytokines. However, developing mAbs targeting these newly identified molecular mechanisms remains costly and technologically challenging. Miniproteins, by comparison, rely on simpler design processes, resulting in lower production costs. Thus, they have the potential to facilitate the rapid development of treatment approaches targeting these novel pathways (Fig. 1).

Moreover, the applications of this technology are not limited to autoimmune diseases. In oncology, for example, mAbs targeting immune checkpoint molecules, such as PD-1/PD-L1 and CTLA4, have substantially improved patient outcomes by enhancing the body’s immune response against tumors. Miniproteins targeting immune checkpoints might help boost immune systems (Fig. 1). Besides, miniproteins could be engineered to target other oncogenic pathways. mAbs have been used to block growth factor signaling in different cancers, such as HER2 in breast cancer, EGFR in colon cancer, and VEGFR in colon, lung, and kidney cancers. It would be interesting to explore whether miniproteins could target these immune checkpoints or growth factor signaling, potentially offering a more accessible and cost-effective alternative to current mAb therapies.

In summary, the study by Berger et al. represents a significant advancement in the field of therapeutic design, demonstrating the potential of computationally engineered miniproteins as effective, orally available alternatives to traditional mAbs. As immune profiling technologies continue to improve, the integration of miniprotein design could accelerate the translation of molecular insights into practical, patient-friendly therapies, ultimately transforming the landscape of modern medicine. Before translating the findings into clinical settings, more studies on different preclinical models will be needed. Clinical studies will be required to validate the exciting findings and explore the full therapeutic potential of miniproteins.
